# The Relationship between Stress and Masticatory Muscle Activity in Female Students

**DOI:** 10.3390/jcm10163459

**Published:** 2021-08-04

**Authors:** Grzegorz Zieliński, Michał Ginszt, Magdalena Zawadka, Katarzyna Rutkowska, Zuzanna Podstawka, Jacek Szkutnik, Piotr Majcher, Piotr Gawda

**Affiliations:** 1 Department of Sports Medicine, Medical University of Lublin, 20-093 Lublin, Poland; magdalena.zawadka@umlub.pl (M.Z.); piotr.gawda@umlub.pl (P.G.); 2 Department of Rehabilitation and Physiotherapy, Medical University of Lublin, 20-093 Lublin, Poland; michal.ginszt@umlub.pl (M.G.); piotr.majcher@umlub.pl (P.M.); 3 Faculty of Pedagogy and Psychology, Maria Curie-Sklodowska University in Lublin, 20-612 Lublin, Poland; kr@psychologsportu.pl; 4 Interdisciplinary Scientific Group of Sports Medicine, Department of Sports Medicine, Medical University of Lublin, 20-093 Lublin, Poland; zuzanna.podstawka@gmail.com; 5 Department of Functional Masticatory Disorders, Medical University of Lublin, 20-093 Lublin, Poland; jacek.szkutnik@umlub.pl

**Keywords:** stress, electromyography, masseter muscle, anterior temporal muscles

## Abstract

The aim of the study was to analyze the relationship between stress measured by the perceived stress scale (PSS-10) questionnaire and masticatory muscle activity. Experimental design assumed the study of healthy young women without temporomandibular disorders, dividing them into three groups depending on the result of the stress level and then comparing these groups in terms of bioelectrical activity of the masticatory muscles. After the exclusion criteria were applied, 63 female students (mean age: 22.3 ± 2.4 years) from Medical University of Lublin were included in the study. The subjects were then divided into 3 groups: with low (*n* = 18), medium (*n* = 18) and high stress level (*n* = 27), according to PSS-10 results. Resting and functional activity of temporalis anterior (TA) and masseter (MM) muscles were measured by electromyograph BioEMG III. There were statistically significant effects of group on the absolute value of asymmetry index (AsI) of TA and MM during maximum voluntary clenching on dental cotton rollers (both *p* = 0.02). Post hoc analysis shows that there were statistically significant differences between medium and high stress groups in AsI TA (*p* = 0.01) and between low and high stress groups in AsI MM (*p* = 0.02). Perceived stress measured by PSS-10 questionnaire seems to be associated with changes in muscular asymmetry in functional clenching activity.

## 1. Introduction

Claude Bernard (1865) found that keeping health depends on maintaining a stable internal environment in the face of changing conditions whereas Cannon (1929) called it “homeostasis” [1]. Selye (1956) used the term “stress” to describe the effects of anything that seriously threatens homeostasis [1]. Actual or perceived threat to an organism is defined as a “stressor”, and the response to a stressor is called a “stress reaction”. Although stress responses have evolved as an adaptive processes, Selye indicated that severe, long-term stress responses can lead to tissue damage and illness [1].

The most common stress-related symptoms affecting mental health can include sleep disorders, depressed mood, sadness, anxiety, irritability, concentration and memory disorders, chronic fatigue syndrome, anorexia and bulimia. It is believed that stress is the cause of the first episodes of depression [2,3]. Somatic disorders connected to stress will include obesity, type 2 diabetes, irritable bowel syndrome, back pain, osteoporosis, dermatological complications, arteriosclerosis, idiopathic hypertension and ischemia of the heart [2]. Stress is linked to neurological disorders tension headaches and migraine [4,5,6]. It is generally accepted that mental stress causes increase muscular tension in different parts of the body [7,8], but the research data are so far inconclusive [9,10]. The American Psychological Association notes that muscle tension is a reflex response to stress and protects body from injury or pain [11]. For patients, tension in the neck and all head area will be especially annoying; it can contribute to tension-type headaches and migraines [4,5,6,11]. Studies have shown higher masticatory muscles activity in people with high levels of stress [2,12]. Increased masticatory muscle tension may be one of the factors predisposing to temporomandibular disorders (TMD) [13]. 

TMD of the masticatory system include issues related to the masticatory muscles, temporomandibular joints and surrounding tissues [14]. By the World Health Organization, temporomandibular disorders are recognized as the third most common dental disease after tooth decay and periodontitis [15]. Due to the complex etiopathology of TMD, four etiological factors can be distinguished: behavioral, social, emotional and cognitive [16]. A number of clinical studies seem to confirm the link between the exacerbation of mastication dysfunction and strong emotional experience, especially in young people in final adolescence and early adulthood [17,18,19,20]. The literature about patients with TMD shows that there is a lack of research on healthy groups and also how stress affects the stomatognathic system. 

The aim of the study was to analyze the relationship between stress measured by the perceived stress scale (PSS-10) questionnaire and masticatory muscle activity. Based on the above-mentioned observations, we hypothesize that perceived stress measured by PSS-10 questionnaire significantly influences the activity of the masticatory muscles. 

## 2. Materials and Methods

### 2.1. Study Population

Eighty-eight women between the ages of 20–30 were invited to the study. The participants were informed about the research objectives and were aware of the possibility of resigning at any time. The presented study was performed in line with the principles of the Helsinki Declaration and was approved by the ethics committee of Medical University of Lublin (approval number KE-0254/93/2020). A written permission was obtained from all the respondents who participated in the study. During the qualification for the study, the Research Diagnostic Criteria for Temporomandibular Disorders (RDC/TMD) clinical examination was performed. All participants were clinically examined based on a two-axis RDC/TMD form by an experienced dentist with a specialization dental prosthetics (the author J.S.). Clinical RDC/TMD assessment was conducted using a standardized clinical protocol including evaluation of patient history, palpation of temporomandibular joints (TMJ) and masticatory muscles, auscultation of joint noises and measurement of mandibular range of motion, according to the RDC/TMD guidelines [21].

Inclusion criteria used in the study were: female gender, age range 20–30 years, four support zones of dental arch, complete dentition and good or very good physical health status determined by the (RDC/TMD) questionnaire. 

The following exclusion criteria were applied during clinical examination: the symptoms of TMDs based on an RDC/TMD examination, neurological disorders within the head and neck area, neoplastic diseases (regardless of type and location), trauma and previous surgical treatment in the head and neck area within the last 6 months before the examination, class II and III according to Angle’s classifications, open bite, crossbite, any inflammatory conditions in oral area, illness or injury in the cervical spine area, orthodontic treatment, possession of dental prostheses (regardless of type), pregnancy.

### 2.2. Study Protocol 

After the exclusion criteria were applied, 63 female students (mean age 22.3 ± 2.4 years) from Medical University of Lublin were qualified for the perceived stress scale questionnaire survey and surface electromyography (sEMG) examinations.

The study was conducted in Department of Functional Masticatory Disorders, Medical University of Lublin, Poland. 

#### 2.2.1. Questionnaire Survey (Perceived Stress Scale PSS-10)

A questionnaire form was used, that is, a psychological scale designed by Cohen et al., the so-called Perceived Stress Scale (PSS-10) [22]. The questionnaire is a self-reported scale to measure the global level of perceived stress with problems and personal events that have occurred during the last month. The scale contains 10 questions concerning various subjective feelings. This scale includes two factors. Factor first (Perceived Helplessness) is made of negatively phrased items (i.e., items 1, 2, 3, 6, 9 and 10, e.g., “In the last month, how often have you been upset because of something that happened unexpectedly?”). The factor second (Perceived Self-Efficacy) is made of positively phrased items (i.e., items 4, 5, 7, and 8, e.g., “In the last month, how often have you felt confident about your ability to handle your personal problems?”) Participants were required to rate how often they felt a certain way over the past month on a five-point Likert scale (0–4), with 0 meaning never, 1 hardly ever, 2 sometimes, 3 quite often, and 4 very often. Calculation of the overall intensity of perceived stress followed a change in the score in positively formulated answers, that is, according to the rule, 0 = 4, 1 = 3, 2 = 2, 3 = 1 and 4 = 0. The overall score obtained after summing up all the answers was then converted into standard units stens: 1–4 stens, 0–13 points, intensity of perceived stress—“Low”; 5–6 stens, 14–19 points-intensity of perceived stress—“Medium”; 7–10 stens, 20–40 stens-intensity of perceived stress—“High” [23]. Sten scores are normalized so that the population standard deviation is 5. 5, and the standard deviation is 2. The Polish version of the PSS-10 was translated and standardized and culturally valid in Polish by Juczyński and Ogińska-Bulik [24]. The studies have suggested that the PSS-10 shows good test–retest reliability, and validity across different populations [25]. The examination was conducted by a psychologist with a PhD degree (the author K.R.).

#### 2.2.2. Assessment of the Masticatory Muscles Activity

The sEMG examination was performed using 8-channel electromyograph BioEMG III, compatible with BioPAK Measurement System (BioResearch Associates, Inc., Milwaukee, WI, USA). Before each examination, an interference test was performed using BioPAK sEMG Noise Test. Moreover, BioPAK Noise Tests were performed for all participants after each measurement. Electric potentials of two pairs of muscles were measured: temporalis anterior (TA) and the superficial part of the masseter muscle (MM). The sEMG examinations were conducted between 8:00 and 12:00 a.m., to minimize the influence of daily fluctuations of muscle activity. The subjects sat on the dental chair; the height of the headrest was adjusted individually to set the head, neck, and torso of the subjects in a straight line. Before placing the surface electrodes (Ag/AgCl with a diameter of 30 mm and a conductive surface of 16 mm—SORIMEX, Toruń, Poland) the skin was cleansed with a 90% ethyl alcohol, over the most bulged muscle mass palpated in contraction, parallel to muscular fibers according to the surface EMG for non-invasive assessment of muscles) program guidelines (SENIAM) [26]. The arrangement of the electrodes symmetrically on the skin covering the examined muscles on both sides in accordance with the course of muscle fibers was preceded by palpation of the muscles during mandibular movements. The sEMG electrodes on the superficial masseter muscle were located along the line from the mandible angle to the inferior border of the zygomatic bone. The electrodes on the temporalis anterior muscle were arranged along a perpendicular line from the superior border of the zygomatic bone to a cranial bone in the projection of the sphenoid bone. Symmetrically two electrodes were glued to the middle of each muscle. 

The arrangement of surface electrodes was carried out by the same physiotherapist (the author M.G.) The reference electrode was placed on the forehead [27]. Before sEMG measurement, all the subjects were instructed in the procedure. The activity of the masticatory muscles (TA, MM) was recorded in the following protocol: during resting mandibular position (10 s), during maximum voluntary clenching (3 clenches of 3 s each with a 2 s break), during maximum voluntary clenching on dental cotton rollers (3 clenches of 3 s with a 2 s break), and during maximum mouth opening (3 abductions of 3 s with a 2 s rest between) [27,28,29]. 

The electromyographic signals obtained during the test were standardly amplified and purified from 99% of the noise scale on a linear scale using the BioPAK digital NoiseBuster filter. Automatic processing of the electromyographic signal, based on the root mean square (RMS) calculation in the BioPAK program, allowed obtaining average measurement values, which were then used to analyze muscle activity. The sEMG examination was always conducted by the same physiotherapist (author G.Z.) The repeatability of the sEMG protocol was tested by duplicate sEMG measurements on the 10 participants. The two independent sEMG measurements were separated by 5 min rest between activities. There were no significant differences (*p* > 0.05) between repeated sEMG records in all analyzed variables (resting mandibular position, maximum voluntary clenching, maximum voluntary clenching on dental cotton rollers, maximum mouth opening).

#### 2.2.3. Asymmetry and Activity Indexes

Asymmetry between left and right masticatory muscle activity was determined quantitatively using the asymmetry index (AsI, unit %, range from 0% to 100%), according to the following equation [30,31]:

AsI = ∑i = 1N|Ri − Li|/∑i = 1N(Ri + Li) × 100(1)

Muscle activity was measured between the anterior part of the temporalis anterior muscle and superficial part of the masseter muscle by means of the activity index (AcI, unit %, range from 0% to 100%), according to the following equation [30,31]:

AcI = ∑i = 1N|MM − TA|/∑i = 1N(MM + TA) × 100(2)

### 2.3. Statistical Analysis

Statistical analysis was carried out using Statistica 13.3 analytics software (TIBCO Software Inc., Palo Alto, CA, USA). Shapiro-Wilk test revealed the data are not distributed normally; therefore, non-parametric tests were used for further statistical analysis of EMG data. Statistical significance was accepted at *p* < 0.05 with all outcome measures reported as median and ranges (minimum and maximum).

A non-parametric Kruskal-Wallis ANOVA test of differences among three stress-groups was conducted and fallowed post hoc analysis. An effect size (ES) was calculated for statistically significant results of non-parametric tests using Eta squared (η^2^). It describes the proportion of the total variability in the data that are accounted for by the effect under consideration. Values of η^2^ in terms of Cohen’s [32] description is considered as large (0.14). medium (0.06). and small (0.01) effects.

## 3. Results

The number of participants in groups of low, medium and high stress was not statistically different. There were no statistically significant differences in age and maximum mouth opening between the groups. Table 1 shows sample structure and comparison of age and range of maximum mouth opening between the groups.

Statistical analysis revealed that there are not statistical differences between the groups in the bioelectrical activity of tested muscles under all conditions (rest, clenching, cotton rollers clenching, mouth opening). Table 2 shows detailed results of the comparisons.

There were statistically significant effects of group on the absolute value of asymmetry index (AsI) of TA and MM muscles during clenching on dental cotton rollers (both *p* = 0.02). Post hoc analysis shows that there were statistically significant differences between medium and high stress groups in AsI TA (*p* = 0.01) and between low and high stress groups in AsI MM (*p* = 0.02). The effect sizes of these results were medium (η^2^ = 0.1 and η^2^ = 0.09, respectively). Details of the comparisons are shown in Table 3.

There was no statistically significant effect of group on the absolute value of activity index (AcI) during all measurements. Table 4 shows detailed results of the comparisons.

## 4. Discussion

The aim of the study was to analyze the relationship of between stress measured by the perceived stress scale (PSS-10) questionnaire and masticatory muscle activity. The analysis showed that there were no significant statistical relationships between psychological stress measured by PSS-10 questionnaire and bioelectrical activity at rest, at occlusion, at occlusion with dental cotton rollers and on maximal opening in female medical students. Statistical analysis demonstrated significant differences in absolute values AsI TA and AsI MM during clenching on dental cotton rollers. Presented study pointed out there is increase of differences in AsI during clenching on dental cotton rollers along with stress level. However, in MM muscles, we observed the biggest differences in subjects with medium stress level during clenching on dental cotton rollers. 

Stress is defined as one of the factors in the development and exacerbation of TMD [16], especially in young people in late adolescence and early adulthood [17,18,19,20]. The selection of the study group due to age is associated with prevalence of mental disorders in the selected group. Research shows that mental health disorders are common in the young population, more common among women than men [33,34]. Determining the level of stress is important to find out if any reduction will affect changes in bioelectrical tension. In the etiology of functional disorders of the masticatory organ, the value of psychological factors, especially stress, neuroticism and depression, was more frequently emphasized in patients with myofascial pain than in those with TMD [18,35]. In determining what the level of disorder was, it was guided by the fact that little of the literature in this subject differentiates the level of stress, and this was only done for patients with TMD [2]. 

A study by Stocka et al. demonstrates that stress measured by PSS-10 questionnaire may reveal or exacerbate symptoms of masticatory dysfunction [2]. Evaluation of muscle activity in groups with different stress level showed a significant difference between the mean value of MM electrical activity and the sum of TA and MM electrical potentials in central occlusion in each group. The sum of the mean values of the electrical potentials of the temporal and masseter muscles on the right side was the highest in the high stress group and differed significantly compared to the other groups [2]. The observations of Stocka et al. are confirmed in the author’s research in the results of activity index in healthy people. A study conducted on rats by Chen et al. indicates the influence of stress on metabolism of masticatory muscles. Authors suggest that stress affects changes in function of mitochondrium [33]. The change in mitochondria may contribute to increased muscle asymmetry during the highest activity which is teeth clenching on the dental cotton rollers. According to the study by Augusto et al., there is a correlation between TMD and variables such as parafunctional habits and perceived stress [20]. The data demonstrated that lack of significant differences between stress and muscle tension levels may be related with the fact that in the study group there were no patients with TMD.

It was observed that depression is not always associated with somatic disorders with tension in the masticatory muscles [34,36,37]. Although etiology of this disorders remains unclear, increasing attention is being paid to the impact of psychological stress on depression [38]. Hypothetically, it can be suggested that, just as in depressive disorders, stress disorders will not be associated with increased bioelectrical tension in the masticatory muscles, as demonstrated in the author’s study.

To sum up, high and medium stress levels, according to the PSS-10 questionnaire, are associated with changes in masticatory muscle symmetry, determined by the AsI during clenching on dental cotton rollers.

These observations may include participation of TA and MM asymmetrical activity in etiology of tension-type headaches, predisposition to TMD and bruxism in people with high stress level. It can accelerate the diagnostics and include stress disorders as a cause of masticatory organ muscle tension. Further research should be continued on a larger, age and sexually diverse groups of respondents. It is also worth conducting research on other ethnic groups to establish a link between psychological factors and bioelectrical parameters of masticatory muscles in different populations.

The presented study has several limitations. Firstly, the diagnostics criteria for TMDs were changed to The Diagnostic Criteria for Temporomandibular Disorders (DC/TMDs) in 2014; however, in the presented study, the previous version was used. There is no validated Polish version of the DC/TMDs so far, and therefore, the RDC/TMDs was used. Secondly, research samples consist of small groups of young females. The effect sizes obtained were small to moderate. Therefore, future studies should involve relevant groups from a larger population including male population and with an extended age. We also suggest comparing the influence of stress on the bioelectrical activity of the masticatory muscles in healthy subjects versus subjects with TMD. Thirdly, to compare the sEMG activity in the field of dentistry, normalization process of the sEMG data during maximum voluntary contraction is often used [39]. However, the results of the presented study were based on the RMS calculation in the BioPAK program, which allowed obtaining average measurement values used to comparison of muscle activity. Moreover, we used activity and asymmetry indices to standardize and compare sEMG results. In addition, both normalized sEMG values and sEMG RMS data were used to compare sEMG results [40,41,42,43].

## 5. Conclusions

Perceived stress measured by PSS-10 questionnaire is associated with a change in muscle asymmetry in functional activity during teeth clenching. Further research should be continued on a larger group of respondents to determine the relationship between psychological factors and bioelectric parameters of the masticatory muscles.

## Figures and Tables

**Table 1:** Group comparisons according to age, maximum mouth opening and number of participants.

	Females					Statistics	
Low stress	*N* = 18					chi 2 = 2.57	
Medium stress	*N* = 18					df = 2	
High stress	*N* = 27					*p* = 0.28	
	Group	Median	Min.	Max.	SD	test	*p*
Age (years)	Low stress	22.00	20.00	27.00	1.75	H = 0.10	0.95
	Medium stress	22.00	19.00	30.00	3.14		
	High stress	22.00	20.00	29.00	2.31		
Mouth opening (mm)	Low stress	51.50	43.00	64.00	5.87	F = 0.17	0.85
	Medium stress	50.50	32.00	62.00	7.53		
	High stress	50.00	41.00	63.00	5.20		

**Table 2:** Comparisons of sEMG (surface electromyography) activity in studied groups.

sEMG (µV)	Low			Medium			High			Statistics	
	Median	Min.	Max.	Median	Min.	Max.	Median	Min.	Max.	H	*p*
REST											
TA-R	1.46	1.06	5.74	1.71	0.80	7.46	1.80	0.59	6.65	0.12	0.94
TA-L	2.00	0.97	6.06	2.22	0.91	6.33	2.15	0.70	9.64	0.23	0.89
TA_Mean_	1.64	1.04	5.22	2.02	0.88	6.90	2.09	0.65	6.27	0.27	0.87
MM-R	1.81	0.93	4.82	1.56	0.73	2.88	1.50	0.86	5.75	2.06	0.36
MM-L	1.83	1.05	5.87	1.45	0.78	5.17	1.66	0.68	3.56	3.09	0.21
MM_Mean_	1.82	0.99	3.94	1.45	0.84	4.01	1.55	0.83	4.66	3.34	0.19
CLENCHING											
TA-R	79.95	10.20	172.10	89.70	11.90	273.30	106.00	28.90	248.70	2.68	0.26
TA-L	99.70	10.90	430.70	104.15	24.00	269.80	121.30	32.10	260.60	1.26	0.53
TA_Mean_	84.85	10.55	277.80	91.00	19.20	218.85	119.65	30.50	254.65	1.48	0.48
MM-R	90.85	10.00	434.10	78.05	9.10	503.40	92.60	5.10	337.90	0.33	0.85
MM-L	94.15	4.60	337.10	97.70	9.00	227.00	104.20	7.00	241.70	0.22	0.90
MM_Mean_	93.03	7.30	385.60	71.00	10.50	352.10	107.45	6.05	288.95	0.03	0.98
CLENCHING ON DENTAL COTON ROLLERS											
TA-R	84.00	21.20	182.60	100.70	37.40	196.60	94.50	35.20	307.20	1.12	0.57
TA-L	80.75	40.00	402.20	119.35	37.60	188.50	109.50	34.70	303.90	0.79	0.67
TA_Mean_	82.38	30.60	261.10	110.68	44.55	170.15	107.65	38.00	295.30	0.83	0.66
MM-R	107.95	54.60	421.50	100.70	37.40	196.60	119.90	11.80	497.50	0.64	0.73
MM-L	110.70	39.40	325.70	119.35	37.60	188.50	117.30	32.00	356.70	0.36	0.83
MM_Mean_	110.80	50.40	365.45	110.68	44.55	170.15	113.05	30.90	427.10	0.20	0.91
MOUTH OPENING											
TA-R	6.75	0.30	13.40	3.95	2.30	10.30	5.50	2.30	17.00	5.03	0.08
TA-L	6.20	2.90	16.50	4.60	2.60	40.00	5.00	2.70	24.40	2.53	0.28
TA_Mean_	6.50	3.35	13.75	4.83	2.45	22.00	6.35	3.00	20.70	3.09	0.21
MM-R	6.20	2.40	46.30	6.15	2.90	17.30	6.20	2.00	26.40	0.26	0.88
MM-L	6.65	1.50	31.20	5.50	2.90	22.00	5.90	2.30	22.40	1.60	0.45
MM_Mean_	6.38	1.95	38.75	5.90	2.90	19.65	6.20	2.30	24.40	0.67	0.71

TA—temporalis anterior; MM—the superficial part of the masseter muscle; R—right; L—left.

**Table 3:** The absolute values of asymmetry index (AsI) in studied groups.

AsI Absolute Value	Low			Medium			High			Statistics	
	Median	Min.	Max.	Median	Min.	Max.	Median	Min.	Max.	H	*p*
REST											
TA	16.59	0.00	40.74	12.06	1.00	59.06	10.87	1.48	60.17	0.32	0.85
MM	6.75	1.44	71.64	11.54	0.58	32.41	11.02	0.00	30.74	0.63	0.73
CLENCHING											
TA	9.84	0.71	55.04	20.18	0.20	56.25	8.22	1.19	40.67	2.96	0.23
MM	11.46	0.75	36.99	14.43	0.70	59.58	15.44	0.70	43.93	3.50	0.17
CLENCHING ON DENTAL COTON ROLLERS											
TA	12.14	0.41	54.04	15.80	0.07	32.26	8.68	0.21	24.48	8.03	0.02 *
											η^2^ = 0.1
MM	6.04	0.73	25.05	13.95	0.94	74.35	16.48	2.14	76.75	7.59	0.02 *
											η^2^ = 0.09
MOUTH OPENING											
TA	13.47	0.00	91.30	10.98	1.27	81.82	17.87	1.01	60.31	1.00	0.61
MM	10.99	2.22	46.48	7.41	0.00	49.43	9.68	0.00	25.19	0.77	0.68

TA—temporalis anterior; MM—the superficial part of the masseter muscle; * Significant difference.

**Table 4:** The absolute values of activity index (AcI) in studied groups.

AcI Absolute Value	Low			Medium			High			Statistics	
	Median	Min.	Max.	Median	Min.	Max.	Median	Min.	Max.	H	*p*
REST											
Right	18.01	1.36	50.68	27.24	0.63	59.43	20.78	0.00	63.19	0.09	0.96
Left	25.69	2.30	36.97	30.74	0.52	61.89	17.58	3.45	57.09	3.10	0.21
Mean	15.21	1.33	44.79	23.09	1.96	56.76	21.57	0.82	41.69	2.01	0.37
CLENCHING											
Right	24.40	0.26	61.87	28.05	0.11	56.56	25.30	0.69	70.00	0.26	0.88
Left	18.63	3.24	77.83	24.90	0.39	77.92	18.48	1.07	68.15	1.26	0.53
Mean	18.60	2.57	71.23	22.31	6.04	72.01	17.79	2.45	66.89	0.60	0.74
CLENCHING ON DENTAL COTON ROLLERS											
Right	21.96	0.63	63.62	18.30	1.18	45.91	26.71	0.29	73.15	0.09	0.96
Left	19.94	0.36	37.23	15.45	2.57	75.91	12.58	1.47	52.30	0.40	0.82
Mean	21.22	1.46	41.74	18.86	1.32	33.29	19.22	1.34	56.41	0.47	0.79
MOUTH OPENING											
Right	22.54	1.05	95.38	13.43	2.44	51.72	21.57	1.14	47.37	2.25	0.32
Left	18.95	1.52	64.64	17.72	2.29	74.67	11.11	0.32	66.01	0.85	0.65
Mean	21.50	0.89	61.02	12.76	3.29	62.96	17.61	1.38	51.01	1.31	0.52

## Data Availability

Not applicable.
